# Elements of Physics

**Published:** 1846-10

**Authors:** 


ART. III.—*Elements of Physics.* By C. F. PESCHEL, Principal of the Royal Military College at Dresden, &c. Translated from the German, with Notes, by E. WEST. Illustrated with 430 Wood-engravings.—*London,* 1845-6. Three Volumes, 12mo, pp. 894.

"THE study of the physical sciences," says the translator, "has become of late almost universal; partly because it has been found well suited to cultivate habits of observation and reasoning, which cannot fail to be of use in the business of life, and partly because the practical application of scientific principles to the details of almost every department of manufacturing and agricultural industry has rendered some acquaintance with natural philosophy and chemistry indispensable." We need not, we trust, now dwell upon the importance of these studies to the youth who is preparing himself for the medical profession; since our opinion of their value has been frequently and strongly expressed. Continued observation, indeed, has only served to convince us yet more decidedly that the *direct* benefits of these pursuits to the medical student, great as these are, are the least of their advantages; and that the philosophic training which they are calculated to afford is, if due pains be taken by the teacher to apply it, the most important result of attention to them. But this result cannot be attained by the acquirement of a mere smattering of acquaintance with physical science, such as may be very useful as regards its practical applications; it requires for its development a closer and more patient study, such as few youths in this country, save those who go through a regular university course, devote to the subject. We quite agree with the translator that, notwithstanding the multiplicity of elementary treatises on physics, there was yet room for another, which, without aiming at being a *popular* introduction, should be sufficiently simple for an intelligent beginner; but which should employ the acquaintance with elementary mathematics, which most schoolboys now gain, as a means of introducing a more scientific method of treatment, such as is at present confined to works of the highest class. And though we cannot doubt, from the excellent manner in which he has translated and edited the work of Peschel, and from the character of the additions which he has made to it, that he was fully competent to produce such a treatise for himself, yet he has not done unwisely, perhaps, in availing himself of one which so admirably fills up the deficiency alluded to.

The first volume, published rather more than a year ago, and containing the physics of ponderable bodies, has been most favorably received by those best qualified to form a judgment of it; and has, we believe, been introduced as a text-book into several of our leading academies. It would, therefore, be superfluous in us to offer any opinion of our own upon its merits; but we may state that a careful examination of the other two volumes, which have recently appeared, has satisfied us that their merit is equal, if not superior, to that of the first; and that the whole book may be most safely recommended to those who desire to attain something more than an elementary knowledge of physical science. It will not supersede the excellent Manual of Dr. Golding Bird, which is more concise in its character and limited in its range, and which is better adapted, therefore, for those who can spare but a small modicum of time for such pursuits, or who are, unfortunately, not sufficiently well grounded in mathematics to avail themselves of the higher advantages of the treatise before us. But we trust the day is not far distant, when every medical student, who aims at taking a respectable stand in his profession, shall have made himself fully master of Peschel's 'Elements of Physics,' even if he proceed no further.

